# Gray matter structures associated with neuroticism: A meta‐analysis of whole‐brain voxel‐based morphometry studies

**DOI:** 10.1002/hbm.25395

**Published:** 2021-03-11

**Authors:** Xiqin Liu, Han Lai, Jingguang Li, Benjamin Becker, Yajun Zhao, Bochao Cheng, Song Wang

**Affiliations:** ^1^ The Clinical Hospital of Chengdu Brain Science Institute, MOE Key Lab for Neuroinformation University of Electronic Science and Technology of China Chengdu China; ^2^ State Key Laboratory of Cognitive Neuroscience and Learning & IDG/McGovern Institute for Brain Research Beijing Normal University Beijing China; ^3^ Huaxi MR Research Center (HMRRC), Department of Radiology West China Hospital of Sichuan University Chengdu China; ^4^ College of Teacher Education Dali University Dali China; ^5^ School of Education and Psychology Southwest Minzu University Chengdu China; ^6^ Department of Radiology West China Second University Hospital, Sichuan University Chengdu China

**Keywords:** anisotropic effect size seed‐based d mapping, dACC/mPFC, mental health, meta‐analysis, neuroticism, personality neuroscience, structural magnetic resonance imaging, voxel‐based morphometry

## Abstract

Neuroticism is major higher‐order personality trait and has been robustly associated with mental and physical health outcomes. Although a growing body of studies have identified neurostructural markers of neuroticism, the results remained highly inconsistent. To characterize robust associations between neuroticism and variations in gray matter (GM) structures, the present meta‐analysis investigated the concurrence across voxel‐based morphometry (VBM) studies using the anisotropic effect size signed differential mapping (AES‐SDM). A total of 13 studies comprising 2,278 healthy subjects (1,275 females, 29.20 ± 14.17 years old) were included. Our analysis revealed that neuroticism was consistently associated with the GM structure of a cluster spanning the bilateral dorsal anterior cingulate cortex and extending to the adjacent medial prefrontal cortex (dACC/mPFC). Meta‐regression analyses indicated that the neuroticism‐GM associations were not confounded by age and gender. Overall, our study is the first whole‐brain meta‐analysis exploring the brain structural correlates of neuroticism, and the findings may have implications for the intervention of high‐neuroticism individuals, who are at risk of mental disorders, by targeting the dACC/mPFC.

## Graphical abstract

In this meta‐analysis, we used the anisotropic effect size signed differential mapping (AES‐SDM) to investigate the association between neuroticism and gray matter (GM) structures based on previous whole‐brain voxel‐based morphometry (VBM) studies. Our analysis revealed that neuroticism was consistently associated with the GM structure of a cluster spanning the bilateral dorsal anterior cingulate cortex and extending to the adjacent medial prefrontal cortex (dACC/mPFC). Our findings may shed light on the elucidation of the neural mechanisms that underpin individual variations in neuroticism and the determination of promising intervention targets for neuroticism, which may reduce the risk of anxiety and mood disorders.

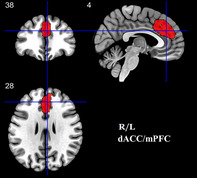

## 1 INTRODUCTION

In the field of psychological research, personality is an important construct and often conceptualized in terms of a universal trait structure (McCrae & Costa, 1997). Neuroticism has long been recognized as one of the most salient and robust higher‐order personality traits across different personality models (Cattell, Eber, & Tatsuoka, 1970; Costa & McCrae, 1985, 1992a, 1992b; Eysenck & Eysenck, 1975; Goldberg, 1990; Zuckerman, 2002). Neuroticism refers to a general tendency towards negative emotional states (e.g., sadness, anxiety, anger, and hopeless) as opposed to positive emotional states (e.g., relaxed, calm, and happy; Costa & McCrae, 1992a; DeYoung, 2015; Widiger, 2009). It plays a central role in both the dominant Five‐Factor Model (FFM) and Eysenck's three‐factor model of personality, which have been operationalized in the Revised NEO Personality Inventory or the NEO Five‐Factor Inventory (NEO‐PI‐R and NEO‐FFI; Costa & McCrae, 1992a, 1992b), and the Eysenck Personality Questionnaire (EPQ) (Eysenck & Eysenck, 1975), respectively. Neuroticism is also termed as emotional stability in the FFM (Goldberg, 1990; McCrae & Costa, 1987). While neuroticism is found to be substantially heritable (heritability estimates: 40–50%; Lahey, 2009; van den Berg et al., 2014), individual differences in neuroticism evolve from the interactions between genes and environment (Canli, 2008; McCrae & Costa, 1997; Ormel et al., 2013a).

Individuals with high neuroticism are more likely to display increased sensitivity to negative or punishment‐signaling cues in the environment (Gray & McNaughton, 1996; Pickering & Gray, 2001), interpret benign situations as threatening (Schneider, 2004) and self‐generate negative affect (Perkins, Arnone, Smallwood, & Mobbs, 2015). Because of the increased reactivity towards negative cues, higher levels of experienced distress and biased cognitive processing, neuroticism may lead to unfavorable life outcomes (Costa & McCrae, 1992a; Hankin, Fraley, & Abela, 2005; Martin, 1985; Tackett & Lahey, 2017). For instance, individuals in the general population who score high on neuroticism are susceptible to negative affect (Costa & McCrae, 1980; Larsen & Ketelaar, 1989, 1991), anxiety and depression symptoms (Hunt, Slade, & Andrews, 2004; Jylhä & Isometsä, 2006; Saklofske, Kelly, & Janzen, 1995), loneliness (Atak, 2009; Kong et al., 2015) and substance abuse (Dubey, Arora, Gupta, & Kumar, 2010; Malouff, Thorsteinsson, Rooke, & Schutte, 2007). Furthermore, meta‐analyses have suggested that neuroticism represents a general risk‐factor for a range of mental disorders including mood disorders, anxiety disorders, eating disorders, substance use disorders, and schizophrenia (Cassin & von Ranson, 2005; Jeronimus, Kotov, Riese, & Ormel, 2016; Kotov, Gamez, Schmidt, & Watson, 2010; Malouff, Thorsteinsson, & Schutte, 2005). The vulnerability model suggests that neuroticism may lead to common mental disorders through processes including a negative bias in attention, interpretation and recall of information; stressful event generation; increased emotional reactivity; and ineffective coping (Ormel et al., 2013b). Moreover, neuroticism is also a stable predictor of physical health problems including hypertension, cardiovascular diseases, and diabetes (Lahey, 2009; Smith & MacKenzie, 2006). Conversely, lower levels of neuroticism—or higher emotional stability—are associated with higher quality of life (Lynn & Steel, 2006; Ozer & Benet‐Martínez, 2006), increased subjective well‐being (DeNeve & Cooper, 1998; Steel, Schmidt, & Shultz, 2008), higher interpersonal relationship satisfaction (Gattis, Berns, Simpson, & Christensen, 2004; Karney & Bradbury, 1995), and greater occupational success (Li et al., 2018; Ozer & Benet‐Martínez, 2006). Given the profound public health significance, especially as a robust risk factor for the development of anxiety and mood disorders (Kendler, Kuhn, & Prescott, 2004; Lahey, 2009), neuroticism has been proposed as a target for clinical diagnostics and intervention (Barlow, Sauer‐Zavala, Carl, Bullis, & Ellard, 2014). However, despite the longstanding importance of neuroticism in personality models and its increasingly recognized relevance for mental and physical health, the brain systems that underlie individual differences in neuroticism have not been robustly determined.

In the past two decades, an emerging field of research (i.e., personality neuroscience) has combined trait approaches with neuroimaging techniques, particularly magnetic resonance imaging (MRI), to map brain regions associated with individual differences in specific traits (Avinun, Israel, Knodt, & Hariri, 2020; Canli, Sivers, Whitfield, Gotlib, & Gabrieli, 2002; Delaparte et al., 2019; DeYoung et al., 2010; Hyatt et al., 2019; Markett, Montag, & Reuter, 2018; Pan et al., 2021; Toschi, Riccelli, Indovina, Terracciano, & Passamonti, 2018; Wang et al., 2018; Wang et al., 2020). In this context, neuroticism is of particular interest because the identification of the underlying neural substrates may contribute to describing a brain‐based predisposition toward psychopathology (Biederman et al., 2001; Khan, Jacobson, Gardner, Prescott, & Kendler, 2005; Ormel et al., 2013b; Watson & Clark, 1992). In particular, an increasing number of studies have investigated brain structural correlates underlying individual differences in neuroticism by means of structural MRI (sMRI; e.g., Avinun et al., 2020; Blankstein, Chen, Mincic, McGrath, & Davis, 2009; Delaparte et al., 2019; DeYoung et al., 2010; Du et al., 2015; Hyatt et al., 2019; Jackson, Balota, & Head, 2011; Kapogiannis, Sutin, Davatzikos, Costa, & Resnick, 2013; Wright et al., 2006). These studies examined the relationship between neuroticism and gray matter (GM) morphometry in healthy subjects using indices such as volume, concentration, cortical thickness or cortical surface area. Findings from these studies suggested that individual differences in neuroticism were primarily associated with GM variations in prefrontal regions, such as the medial prefrontal cortex (mPFC) and orbitofrontal cortex (OFC; Delaparte et al., 2019; DeYoung et al., 2010; Kapogiannis et al., 2013; Wright et al., 2006), as well as anterior cingulate cortex (ACC; Blankstein et al., 2009; Du et al., 2015). Some studies additionally reported associations between neuroticism and GM variations in limbic regions (e.g., amygdala and hippocampus), but findings regarding limbic regions remained generally inconsistent. For example, while the first voxel‐based morphometry (VBM) study of neuroticism revealed that neuroticism was negatively correlated with GM concentration (GMC) in the right amygdala (Omura, Constable, & Canli, 2005), subsequent studies examining GM volume (GMV) found no relationship between neuroticism and amygdala volume (Cremers et al., 2011; Wright et al., 2006). Also, it has been reported that neuroticism was positively correlated with GMV of the hippocampus and the right parahippocampal gyrus (PHG; Kapogiannis et al., 2013), whereas other studies found that neuroticism was not significantly correlated with the GMV of hippocampus or PHG using region of interest (ROI) approaches (Du et al., 2015; Jackson et al., 2011). These inconsistent findings may be accounted for by factors such as different sample sizes, heterogeneity with regard to age and gender distributions, and different measurement and analytical approach (Kapogiannis et al., 2013; Koelsch, Skouras, & Jentschke, 2013; Lu et al., 2014; Yang et al., 2017). Hence, to reconcile the inconsistent findings from individual studies and to better understand the neural substrates underlying neuroticism, the present study employed a quantitative meta‐analytic approach to synergize results from previous studies examining GM correlates of neuroticism.

To this end, we performed a coordinate‐based meta‐analysis of sMRI studies that used whole‐brain VBM approaches to identify brain regions in which GMV/GMC variations correlated with individual differences in neuroticism in healthy subjects. For the meta‐analytic examination of these previous studies, the anisotropic effect size version of seed‐based d mapping (AES‐SDM) method was applied given that it has been widely used in neuroimaging meta‐analyses to assess GM abnormalities in patients (Kolesar, Bilevicius, Wilson, & Kornelsen, 2019; Norman et al., 2016; Radua & Mataix‐Cols, 2009; Wang, Cheng, Luo, Qiu, & Wang, 2018), as well as GM correlates in healthy population (Lai et al., 2019). AES‐SDM adopts a quantitative whole‐brain meta‐analytic method with strict selection of peak coordinates and unbiased inclusion of null findings, thus facilitating unbiased results (Radua et al., 2012; Radua & Mataix‐Cols, 2009). Given that previous studies reported that age or gender influenced personality‐brain associations (Blankstein et al., 2009; Cremers et al., 2011; Nostro, Müller, Reid, & Eickhoff, 2017), additional meta‐regression analyses were conducted to explore a potential confounding effect of these variables on the meta‐analytically determined GM correlates of neuroticism. Notably, our meta‐analysis was limited to VBM studies on neuroticism, as current whole‐brain studies using other GM morphometric measurement are still insufficient. Additionally, we focused solely on the GM findings in healthy people since there exist significant difference between patients and healthy people on both behavioral and brain level.

## 2 MATERIALS AND METHODS

### 2.1 Literature search

The literature on brain structural studies of neuroticism was systematically searched up to July 31, 2020 from databases including PubMed, Scopus and Web of Science. The following keywords were used: “Neuroticism” AND (“MRI,” OR (“magnetic resonance imaging”), OR (“gray matter”), OR (“VBM”), OR (“voxel based morphometry”), OR (“SBM”), OR (“surface based morphometry”), OR (“cortical thickness”), OR (“surface area”), OR(“cortical folding”), OR (“brain structure”), OR (“neuroimaging”), OR (“brain imaging”), OR (“imaging”)). Broad search terms were used to prevent missing any relevant studies. In addition, we inspected reference lists of relevant reviews to identify further eligible articles. Note that we also used “emotional stability” and the same imaging keywords to identify relevant VBM studies and there were no eligible studies, thus the subsequent analyses and results focused on studies found with the keyword “neuroticism.”

### 2.2 Inclusion and exclusion criteria

The inclusion criteria for the original studies were as follows: (a) healthy subjects were examined; (b) focused on neuroticism (emotional stability) as primary research variable; (c) used VBM analysis; (d) reported whole‐brain results and regional GM correlates of neuroticism (including nonsignificant results); and (e) reported results in terms of coordinates provided in Talairach or Montreal Neurological Institute (MNI) space.

Studies were excluded if they: (a) were non‐empirical studies (e.g., review, meta‐analysis, meeting abstract); (b) were not published in English; c) reported insufficient data (e.g., sample size, etc.) even after we contacted the corresponding authors via email; (d) only reported ROI results.

### 2.3 Data selection and extraction

**FIGURE 1:**
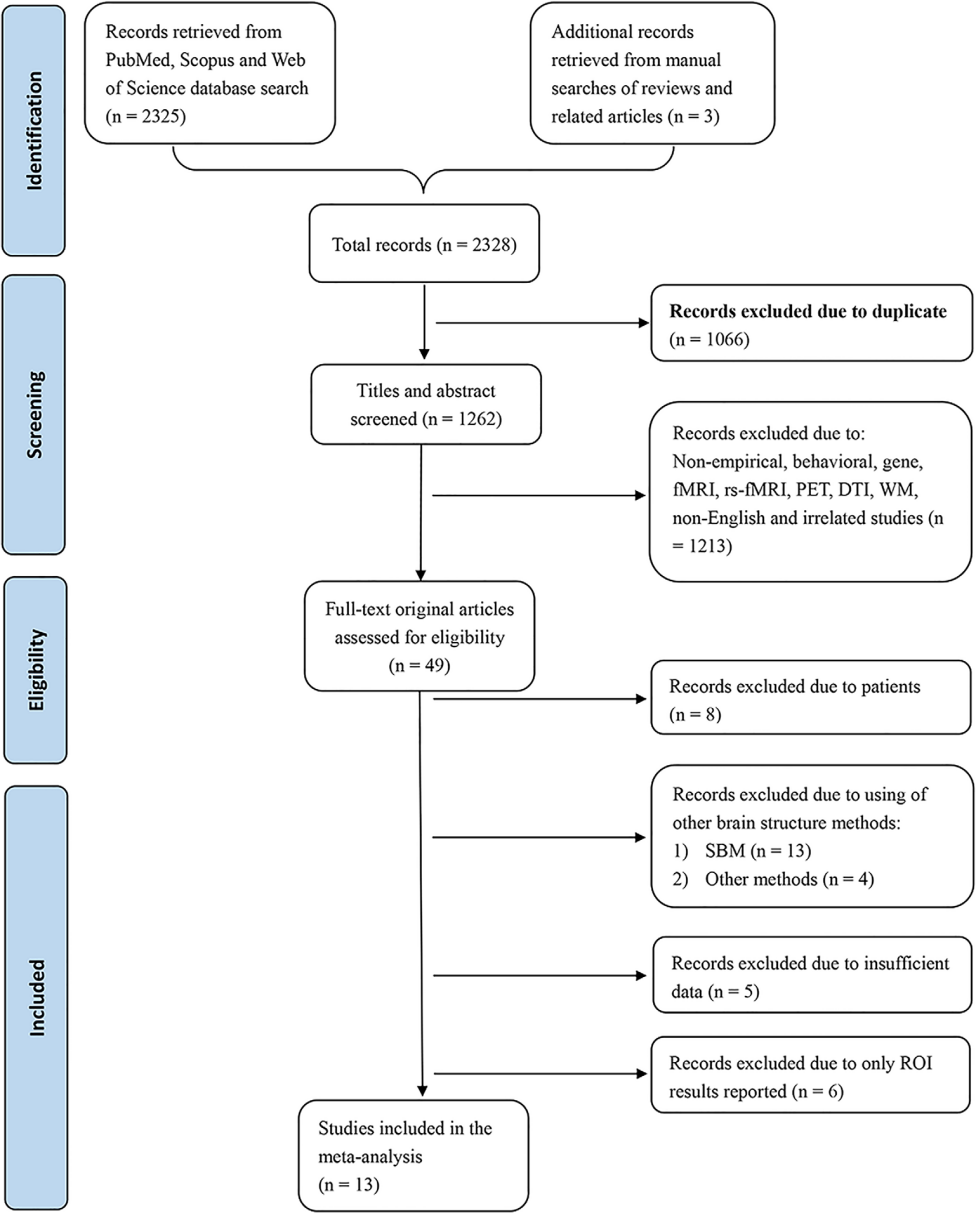
PRISMA flow diagram of data selection in the current meta‐analysis. fMRI, functional magnetic resonance imaging; rs‐fMRI, resting‐state fMRI; PET, positron emission tomography; DTI, diffusion tensor imaging; WM, white matter; SBM, surface‐based morphometry; ROI, region of interest

Two authors (H. L. and X. Q. L) were responsible for screening and assessing each article independently. Consistency was assessed by a third author (S. W.). Figure 1 illustrates the integrated data selection steps according to the Preferred Reporting Items for Systematic Reviews and Meta‐Analyses statement (Moher, Liberati, Tetzlaff, &Altman, 2009). For each study included in the meta‐analysis, peak coordinates of significant GM correlates of neuroticism at the whole‐brain level and other basic information (e.g., sample size, gender ratio, mean age, etc.) were independently extracted by two authors (H. L. and X. Q. L).

### 2.4 Meta‐analysis of VBM studies

The AES‐SDM software, version 5.15 (https://www.sdmproject.com/software/), was used to identify brain regions where the GM structure was reliably correlated with neuroticism. According to the methods proposed by Radua and Mataix‐Cols (2009) and Radua et al. (2012), the meta‐analysis included the following steps. First, the reported peak coordinates and *t*‐values from each included study were extracted to recreate a map of effect size by means of a Gaussian kernel. Second, voxels from these effect size brain maps were randomly permuted to create voxel‐wise Monte Carlo brain maps. Finally, the meta‐analysis effect‐size mean map weighted by sample size, intra‐study variability and inter‐study heterogeneity was compared to a null distribution created with a permutation test. As recommended in previous studies (Lai et al., 2019; Radua et al., 2012; Yao et al., 2017), 20 Monte Carlo randomizations were used to obtain more reliable and stable results from the meta‐analysis. A standard SDM threshold (voxel‐wise *p* < .005, SDM‐Z > 1 and cluster size >10 voxels), which has been verified to effectively balance false positive and negative results (Radua et al., 2012), was applied for the main analyses.

To assess the robustness of the findings, a jackknife sensitivity analysis was performed by iteratively repeating the same meta‐analysis using a leave‐one‐out approach (Radua et al., 2012; Radua & Mataix‐Cols, 2009). Egger's test was conducted to assess a potential publication bias, and a *p*‐value larger than .05 was considered to reflect a lack of a publication bias (Egger, Smith, Schneider, & Minder, 1997; Sterne & Egger, 2001). Funnel plots were additionally created for significant meta‐analytic clusters to examine whether the findings might have been driven by individual studies.

Heterogeneity analyses with Q statistics were carried out to examine inter‐study heterogeneity of individual clusters (Radua et al., 2012; Radua & Mataix‐Cols, 2009). Meta‐regression analyses were conducted to identify potential demographic effects (i.e., gender and age) on the association between neuroticism and GM structures. Given the exploratory nature of meta‐regression analysis, we used a more stringent voxel‐level threshold (voxel‐wise *p* < .0005, z > 1 and cluster size >10 voxels) to decrease false positive findings (Radua et al., 2012; Radua & Mataix‐Cols, 2009).

### 2.5 Functional characterization of identified cluster

To facilitate the functional interpretability of identified cluster, we used *Neurosynth* database (http://www. neurosynth.org) for data‐driven characterization. *Neurosynth* is an automated brain mapping database based on >14,000 functional MRI studies and can be queried for the functional decoding of identified clusters in MNI space (Yarkoni, Poldrack, Nichols, Van Essen, & Wager, 2011). We extracted the psychological terms of which the meta‐analysis map showed the largest correlations with the identified cluster as did in previous studies (e.g., Montag et al., 2019; Xu et al., 2020; Xu, Liu, Qin, Jiang, & Yu, 2020). We further showed seed‐based resting‐state functional connectivity maps obtained by *Neurosynth* for reference, given that the brain‐personality association has been proposed to be characterized on the network level (Markett et al., 2018).

## 3 RESULTS

### 3.1 Included studies and sample characteristics

A total of 13 studies were included in the meta‐analysis, corresponding to a total of 2,278 healthy subjects (1,275 females, 29.20 ± 14.17 years old; see Figure 1 and Table 1).

**TABLE 1:** Details of the studies included in the meta‐analysis

Included study	Sample size	Ratio F/M	Mean age (*SD*)	Scales	Scanner/FWHM (mm)	Nuisance covariate	Statistical analysis/*p*‐value corr	Outcome
Cremers et al., 2011 ^b^	65	42/23	40.5 (9.7)	NEO‐FFI	3 T/8	Age, scan center, TGMV	GLM/ *p* < .05, FWE corr & *p* < .001, uncorr	LH MTG, −64,43,‐2, pos RH SMA, 13,20,68, neg
Du et al., 2015 ^b^	298	158/140	19.94 (1.26)	NEO‐PI‐R	3 T/−	Gender, age, TGMV	Multiple regression/*p* < .05, non‐stationary cluster corr	RH dACC, 3,30,36, pos
Hu et al., 2011 ^b^	62	31/31	26.6 (4.5)	NEO‐FFI	1.5 T/8	Gender, age, TGMV	GLM/ *p* < .05, FWE corr	No correlation
Kapogiannis et al., 2013 ^b^	87	42/45	72 (7.7)	NEO‐PI‐R	1.5 T /12	TIV, years of education	GLM/*p* < .05, FWE corr	RH LG, 14,‐66,2, pos RH FG, 53,‐58,‐24, pos RH MOG, 29,‐98,1, pos RH PCG, 36,‐27,66, pos RH Calcarine, 10,‐82,16, pos LH IOFG, 42, 38, −23, neg RH Rol Opp, 70, −7,12, neg RH MFG, 52,46,15, neg RH PHG, 20,2,‐30, ne RH MTG, 59,1,‐26, neg
Koelsch et al., 2013 ^b^	59	34/25	24.15 (2.40)	NEO‐FFI&NEO‐PI‐R	3 T/4	Gender, age, TIV	Correlation/ *p* < .05, FWE corr	No correlation
Liu et al., 2013 ^b^	227	168/59	25.8 (8.35)	NEO‐FFI	1.5 T/3 T/8	Gender, age, scanner type	GLM/ *p* < .05, FWE corr	No correlation
						Other four traits		
Lu et al., 2014 ^b^	71	37/34	22.35 (1.5)	EPQ‐RSC	3 T/8	Gender, age, TIV	GLM/ *p* < .05, AlphaSim corr	RH cerebellum, 8,‐41, ‐14, pos LH SFG, −20,13,56, neg
Nostro et al., 2017 ^b^	364	182/182	29.1 (3.45)	NEO‐FFI	3 T/8	Gender, age, TIV	GLM/ *p* < .05, FWE corr	No correlation
Omura et al., 2005 ^a^ ^,^ ^b^	41	22/19	23.8 (5.4)	NEO‐PI‐R	3 T/12	Gender, age	GLM/ *p* < .001, uncorr	LH SPL, 21,‐68,50, neg RH AG, 62,‐55,34, neg
Taki et al., 2013 ^b^	274	161/113	51.2 (11.8)	NEO‐PI‐R	0.5 T/8	Gender, age, TIV	GLM/ *p* < .05, FWE corr	No correlation
Xu et al., 2020 ^b^	274	148/126	22.8 (2.4)	EPQ	3 T/4	Gender, age, years of education, trait anxiety, depression, harm avoidance	Multiple regression/*p* < .05, non‐stationary cluster corr	No correlation
Yang et al., 2017 ^b^	356	200/156	20.00 (1.32)	NEO‐PI‐R	3 T/10	Gender, age, TGMV, intelligence, family income, education years of parents	Multiple regression/ *p* < .05, non‐stationary cluster corr	RH MFG, 12,51,20, pos
Zou et al., 2018 ^b^	100	50/50	21.91 (2.29)	EPQ‐RSC	3 T/6	Gender, age	GLM/*p* < .05, FWE corr	No correlation

*Note:* For studies including both ROI and whole‐brain results, only whole‐brain results were included in the meta‐analysis. All the peaks coordinates were reported in MNI space. And all Images were processed using VBM toolbox within the SPM.

Abbreviations: AG, angular gyrus; corr, correction; dACC, dorsal anterior cingulate cortex; EPQ‐RSC, Eysenck Personality Questionnaire‐Revised Short Scale for Chinese; F, female; FG, fusiform gyrus; FWE, family‐wise error; FWHM, full width at half maximum; GLM, general linear model; IOFG, inferior orbital frontal gyrus; LG, lingual gyrus; LH, left hemisphere; M, male; MFG, middle frontal gyrus; MOG, middle occipital gyrus; MTG, middle temporal gyrus; neg, negative correlation; NEO‐FFI, NEO Five Factor Inventory; NEO‐PI‐R, Revised NEO Personality Inventory; PCG, precentral gyrus; pos: positive correlation; PHG, parahippocampal gyrus; RH, right hemisphere; Rol Opp, Rolandic operculum; SFG, superior frontal gyrus; SMA, supplemental motor area; SPL, superior parietal lobule; TGMV, total gray matter volume; TIV, total intracranial volumes; uncorr, uncorrection.

^a^ Studies focusing on the gray matter concentration analysis.

^b^ Studies reported significant results, or nonsignificant results.

As shown in Figure 1, among the 2,328 candidate articles, 1,066 duplicates were first rejected. Then, 1,213 studies were excluded from the remaining 1,262 studies after screening of titles and abstracts. Subsequently, 49 full‐text original articles were further evaluated for eligibility. Next, eight studies were excluded from the meta‐analysis due to using patients as participants (Benedict et al., 2008, 2013; Bonet et al., 2019; Duron et al., 2014; Li et al., 2018; Mahoney, Rohrer, Omar, Rossor, & Warren, 2011; Nickson et al., 2016; Weber et al., 2012). Additionally, 17 studies were excluded from the meta‐analysis due to the use of non‐VBM analytic approaches for brain structure such as surface‐based morphometry (SBM; Bjørnebekk et al., 2013; Castagna, 2019; Delaparte et al., 2019; Hyatt et al., 2019; Jackson et al., 2011; Opel et al., 2018; Owens et al., 2019; Privado, Roman, Saenz‐Urturi, Burgaleta, & Colom, 2017; Riccelli et al., 2017; Schutter, Koolschijn, Peper, & Crone, 2012; Schutter, Meuwese, Bos, Crone, & Peper, 2017; Sweeney, Tsapanou, & Stern, 2019; Zhu, Wang, Cao, Zhang, & Qiu, 2020), and other volumetric measures (Chan et al., 2016; Giannakopoulos et al., 2020; DeYoung et al., 2010; Hu et al., 2017). Moreover, five studies with insufficient data such as not reporting peak coordinates or sample size (Knutson, Momenan, Rawlings, Fong, & Hommer, 2001; Kühn et al., 2020; Kunz, Reuter, Axmacher, & Montag, 2017; Montag et al., 2013; Tuerk et al., 2016) were excluded. Finally, six VBM studies were excluded due to only reporting ROI results (Blankstein et al., 2009; Hermann, Bieber, Keck, Vaitl, & Stark, 2014; Joffe et al., 2009; Kong et al., 2015; Kong et al., 2019; Wei et al., 2015). Thus, a total of 13 eligible studies were finally included in the AES‐SDM analysis (see Table 1).

### 3.2 Meta‐analysis of GM

#### 3.2.1 Core brain regions correlated with neuroticism

**FIGURE 2:**
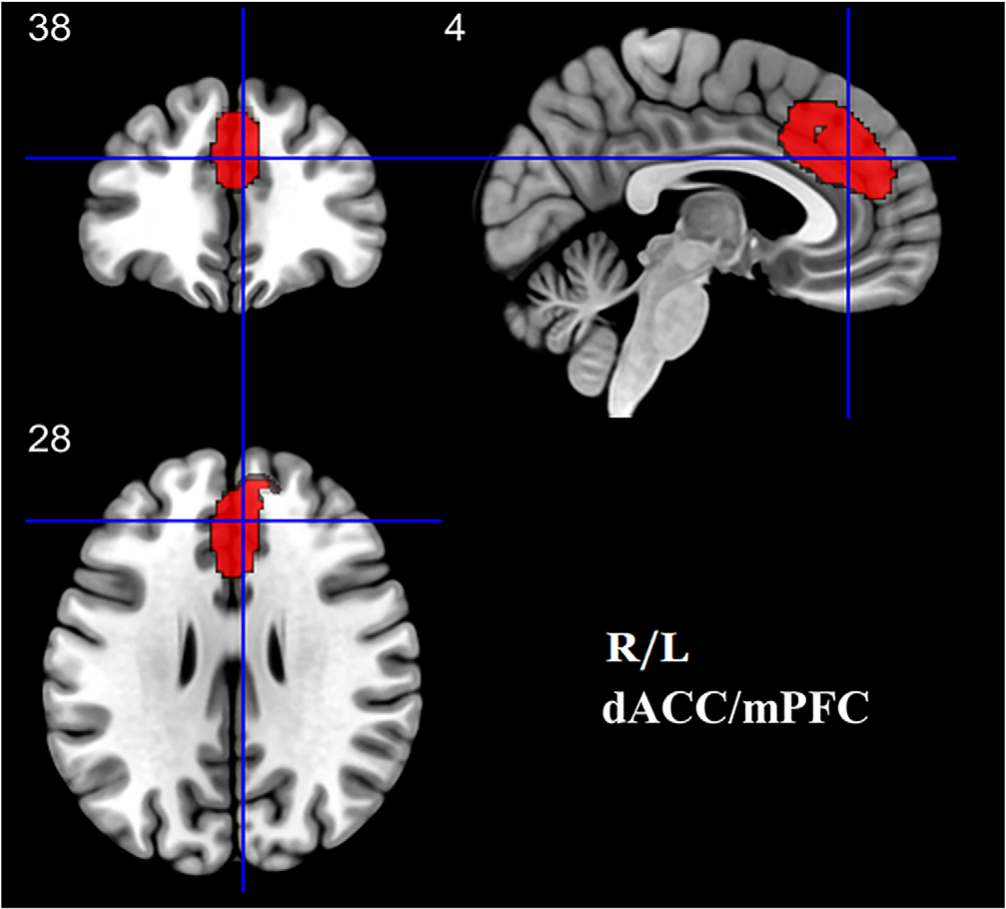
Brain regions showing positive correlation with neuroticism in the meta‐analysis. Clusters were displayed at voxel‐wise *p* < .005, z > 1 and cluster size >10 voxels

The meta‐analysis revealed that neuroticism was positively correlated with the GM structure of a cluster in the bilateral dorsal ACC extending to the mPFC (dACC/mPFC; 1,428 voxels; MNI coordinates: 4, 38, 28; peak *Z* value = 1.714; Brodmann area 32; see Figure 2). There was no negative association between neuroticism and the GM structures.

#### 3.2.2 Reliability of main findings

The Jackknife sensitive analysis revealed that the neuroticism‐GM association in the dACC/mPFC was preserved throughout all the 13 study combinations (see Table S1), indicating that the result was highly reproducible. The funnel plot was found to be roughly symmetric and the Egger's test did not yield significant result (*p* > .05, Bonferroni corrected), suggesting that there was no publication bias in the identified cluster.

#### 3.2.3 Heterogeneity and meta‐regression analysis

The heterogeneity analysis exhibited significant inter‐study variabilities of the included VBM studies in a number of regions including bilateral median cingulate gyri/medial superior frontal gyrus, right lingual gyrus, right inferior frontal gyrus, right rolandic operculum, and right middle frontal gyrus (see Table S2).

To control for potential effects of gender and age, the meta‐regression analyses were conducted and the results showed no association between GM correlates of neuroticism and gender ratio or mean age, suggesting that the association of neuroticism with GM structure in the dACC/mPFC was not modulated by gender or age. Further validation analysis was performed to rule out the confounding effect of age by repeating the main analysis after removing two studies with elderly subjects (Kapogiannis et al., 2013; Taki et al., 2013), and the results remained robust (see Table S3).

#### 3.2.4 Mapping onto the large‐scale networks of the brain

To further map dACC/mPFC identified in the meta‐analysis onto functional networks of the brain, we used Shirer, Ryali, Rykhlevskaia, Menon, and Greicius's (2012) large‐scale networks based on whole‐brain functional connectivity analysis (http://findlab.stanford.edu/functional_ROIs, *Stanford University*, *Palo Alto, CA*). Results showed that the majority of voxels mapped onto the anterior salience network and dorsal default mode network. There were also overlapping voxels in the left and right executive control network (see Table 2).

**TABLE 2:** Spatial overlap between dACC/mPFC and Shirer et al.'s (2012) functional connectivity brain networks

Network	# Of voxels (Total)	# Of voxels (overlap)	Percentage (%)
Anterior salience network	1,428	630	44.12
Auditory		0	0
Basal ganglia		0	0
Dorsal DMN		376	26.33
Higher visual		0	0
Language		0	0
Left ECN		29	2.03
Sensorimotor		0	0
Posterior salience network		0	0
Precuneus		0	0
Primary visual		0	0
Right ECN		35	2.45
Ventral DMN		0	0
Visuospatial		0	0
All		1,070	74.93

Abbreviations: DMN, default mode network; ECN, executive control network.

#### 3.2.5 Functional characterization of dACC/mPFC

**FIGURE 3:**
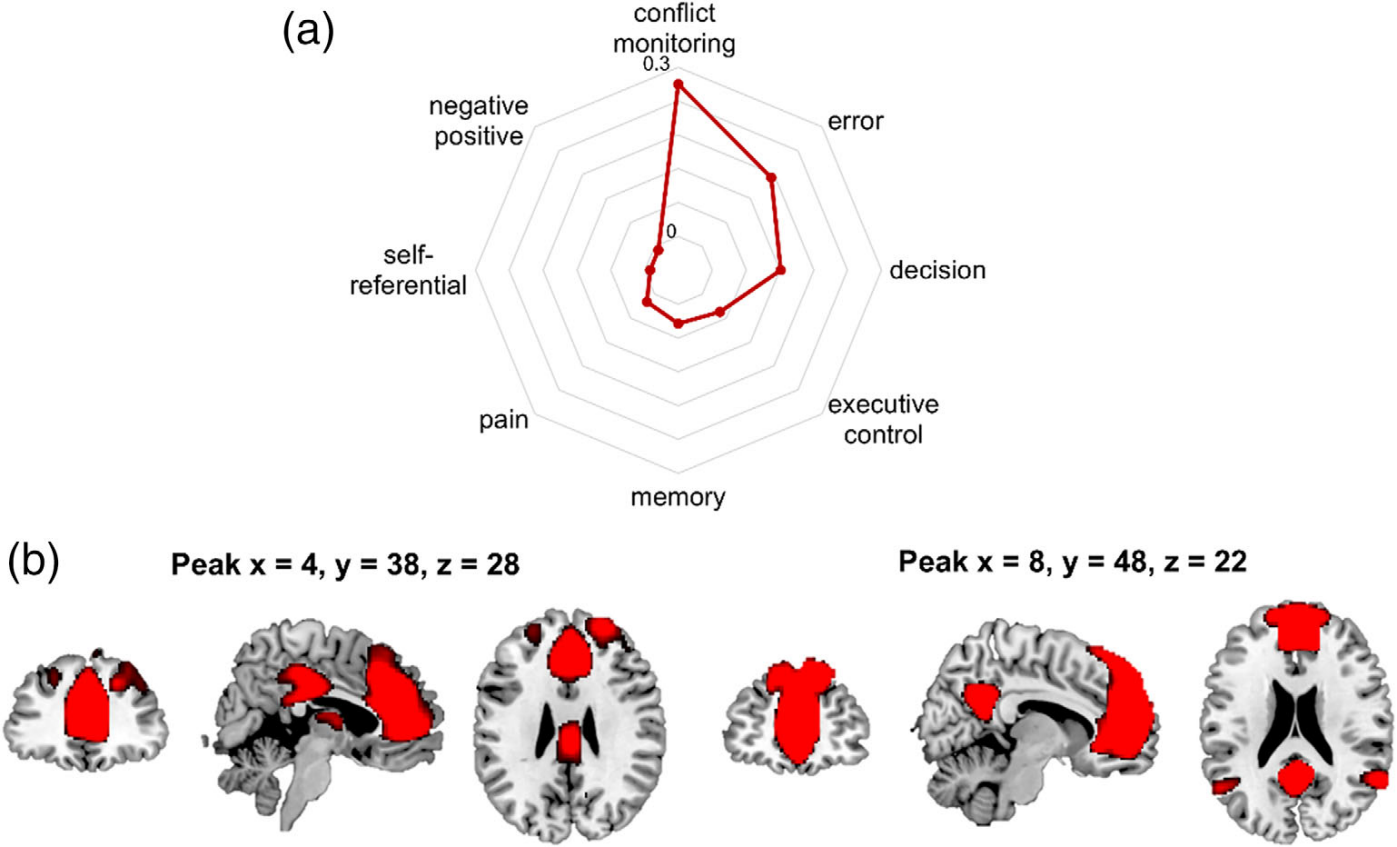
(a) Functional characterization of dACC/mPFC. The statistics in the circular plots are *r* values from Pearson correlations. In the current *Neurosynth* framework, the *r* values reflect the correlation across all voxels between two maps. (b) Resting‐state functional connectivity networks centered around the two peak locations as seed regions. Connectivity maps implicate mPFC, PCC/precuneus, angular gyrus as the most likely projection side of the dACC/mPFC cluster. Data are taken from *Neurosynth*

We showed a radar chart depicting the functional profiles of dACC/mPFC defined by *NeuroSynth* (see Figure 3a). Terms with similar functions were merged into one and retained for the larger correlations. For example, conflict monitoring was the combination of conflict and monitoring from the decoding table. It is notable that the exact *r* values are of less importance and may be difficult to interpret according to the instruction on *Neurosynth* (https://neurosynth.org/decode/), and what is more important is that the extracted psychological terms are of higher correlations relative to others. As shown, the functional decoding of dACC/mPFC was strongly associated with cognitive, interoceptive, and emotional processes such as conflict monitoring, pain, self‐referential processing, and negative/positive affective processing.

The functional connectivity maps (*r* > 0.2, see Figure 3b) implicated several regions such as mPFC, posterior cingulate cortex (PCC)/precuneus, and angular gyrus as the potential projection areas, which constitute the DMN (Raichle et al., 2001).

## 4 DISCUSSION

To the best of our knowledge, the present study is the first meta‐analytic investigation of brain structural correlates of neuroticism, and thus may contribute to the determination of study‐invariant and robust associations between neuroticism and brain structure. Despite heterogenous findings with respect to the brain structural correlates of neuroticism in previous VBM studies, our meta‐analysis identified a robust association between higher levels of neuroticism and greater bilateral dACC/mPFC GM structure. Further examination of the identified cluster revealed spatial distribution in the anterior salience network, dorsal DMN and ECN from the large‐scale functional connectivity networks (see also Table 2). Our finding may thus shed light on the elucidation of the neural mechanisms that underpin individual variations in neuroticism and the determination of promising intervention targets for neuroticism, which may reduce the risk of anxiety and mood disorders.

Neuroticism is typified by the propensity to experience negative emotions (John, Naumann, & Soto, 2008; Kalokerinos et al., 2020; Widiger, 2009). Early electrophysiological studies on neuroticism suggest a hyperarousal of limbic structures, and amygdala in particular, during automatic responses to emotional stimuli such as threats or danger (Cheng, Richards, & Helmstetter, 2007; DeYoung & Gray, 2009; Norris, Larsen, & Cacioppo, 2007). This view has been challenged by emerging evidence from neuroimaging studies, suggesting that neuroticism represents a failure of high‐order cortical interpretation (i.e., by areas of the PFC), rather than overactive emotion generation processes per se (Castagna, 2019; Silverman et al., 2019). Functional MRI (fMRI) studies have found no association between neuroticism and amygdala activation in negative emotional face processing (Cremers et al., 2010; Haas, Constable, & Canli, 2008; Servaas et al., 2013; Silverman et al., 2019; Thomas et al., 2011). Instead, neuroticism has been reported to correlate with amygdala‐ACC connectivity (Cremers et al., 2010), and amygdala‐ventromedial PFC connectivity (Silverman et al., 2019) during negative emotion processing. Previous fMRI studies have found that cognitive evaluation of threatening stimuli is associated with decreased amygdala reactivity and concomitantly increased activity in the PFC and ACC, suggesting that these prefrontal regions are involved in downregulating amygdala's activity through conscious reappraisal and evaluation of the emotional responses (Hariri, Bookheimer, & Mazziotta, 2000; Hariri, Mattay, Tessitore, Fera, & Weinberger, 2003). More direct evidence from sMRI studies has demonstrated that there is no relationship between amygdala volume and neuroticism (Avinun et al., 2020; Castagna, 2019; Cremers et al., 2011; Delaparte et al., 2019), but amygdala volume positively predicts neuroticism through an indirect effect of cortical surface area of superior frontal gyrus and rostral middle frontal gyrus, largely overlapping with mPFC (Castagna, 2019). Diffusion tensor imaging studies have also observed that structural dysconnectivity between PFC and amygdala is associated with neuroticism (Xu & Potenza, 2012). These findings together suggest a regulatory role of prefrontal regions in top‐down control of negative emotions in the individual differences in neuroticism.

In the present meta‐analysis study, we observed a positive association between GM structures in the dACC/mPFC and neuroticism, supporting the aforementioned idea that prefrontal regions play a predominant role in accounting for the individual differences in neuroticism. Functional decoding of the identified cluster through *Neurosynth* displayed associations with a variety of processes including cognitive, interoceptive, and affective processing (see Figure. 3a), consistent with large‐scale meta‐analytic studies suggesting that the mPFC, and the dACC in particular, might implement a domain‐general process that is integral to negative affect, pain, and cognitive control (de la Vega, Chang, Banich, Wager, & Yarkoni, 2016; Shackman et al., 2011).

Traditional models claim that dACC/mPFC is specialized for cognitive control and executive functions including conflict monitoring (Botvinick, Braver, Barch, Carter, & Cohen, 2001) and error detection (Botvinick, Cohen, & Carter, 2004; Gehring, Goss, Coles, Meyer, & Donchin, 1993). By providing evidence from physiological, anatomical and functional aspects, Shackman et al. (2011) proposed the “adaptive control hypothesis” arguing that dACC implements adaptive control over negative affect, pain, or cognitive tasks, by integrating information about punishment and threats arriving from striato‐limbic regions, in order to bias responding in face of uncertainty and conflict. This neural account may help shed light on the behavioral and neural profiles of neuroticism. A number of fMRI studies have found a positive correlation between neuroticism and dACC/mPFC reactivity not only toward negative visual stimuli (Canli et al., 2001; Chan, Norbury, Goodwin, & Harmer, 2009; Cremers et al., 2010; Haas, Constable, & Canli, 2008; Servaas et al., 2013), but also to discrepancy detection requiring inhibitory control (Eisenberger, Lieberman, & Satpute, 2005). Event‐related potential studies have also showed that the error‐related negativity (ERN), which is thought to be generated by dACC/mPFC (Dehaene, Posner, & Tucker, 1994) and involved in detecting and correcting errors reflecting sensitivity to potential threat (Gehring, Goss, Coles, Meyer, & Donchin, 2018), is associated with the level of neuroticism (Luu, Collins, & Tucker, 2000). ERN has also been found to be modulated by expectation–outcome uncertainty (Holroyd & Coles, 2002; Jackson, Nelson, & Proudfit, 2015), which may trigger negative affect and anxiety (Shackman et al., 2011). Previous studies suggest that higher levels of neuroticism are associated with an increased intolerance of uncertainty (Carleton, 2016; Sexton, Norton, Walker, & Norton, 2003). Together, increased GM structure in the bilateral dACC/mPFC may be a structural foundation that reflects greater cognitive control efforts in high neurotic individuals over their biased attention and heightened sensitivity to negative emotions, possibly induced by uncertainty and conflict.

On the network level, mapping the identified cluster on the large‐scale brain networks suggested that the majority of voxels mapped onto the anterior salience network. Indeed, the dACC is proposed as a core node of the salience network (Seeley et al., 2007; Uddin, 2015), which is engaged in affective processing of anxiety‐related information (Markett, Jawinski, Kirsch, & Gerchen, 2020). The intrinsic functional connectivity of the dACC within the salience network is suggested to correlate with state anxiety in healthy subjects (Seeley et al., 2007). Moreover, high trait anxiety, which is considered a component of neuroticism (Widiger, 2009), has also been found to be associated with dysfunctional network interactions between regions of the salience network (e.g., dACC) and other networks (Markett, Montag, Melchers, Weber, & Reuter, 2016; Sylvester et al., 2012). Thus, increased GM structure in the dACC/mPFC in neurotic individuals may be one of the neural bases contributing to salience processing.

Moreover, the identified dACC/mPFC also mapped onto the dorsal DMN (see Table 2). As a key hub of DMN, as also reflected by functional connectivity maps obtained by *Neurosynth* (see also Figure 3a), mPFC is engaged in self‐oriented processing such as self‐referential processing (Menon, 2011; Northoff & Bermpohl, 2004) and autobiographical memory (Spreng & Grady, 2010). It has been proposed that neuroticism is driven by an increased tendency for generating self‐referential cognition, especially negative thoughts (Perkins et al., 2015), and recall of negative real‐life events (i.e., autobiographical memory; Denkova, Dolcos, & Dolcos, 2012), which is associated with structural integrity and activity changes in the mPFC (Bernhardt et al., 2014; Smallwood & Schooler, 2015). Further, aberrant DMN activity and mPFC‐ACC connectivity are related to rumination (i.e., the repetitive and negative self‐referential processing) in depression (Hamilton et al., 2011; Young, Mueller, & Tendolkar, 2016), which exhibits strong link with neuroticism (Jeronimus et al., 2016; Kotov et al., 2010). In a treatment study, subjects scored higher on neuroticism showed decreased activity in the dACC/mPFC in response to negative self‐referential processing in a word categorization task after administration of antidepressant (Di Simplicio, Norbury, & Harmer, 2012) than those receiving placebo. Lesion studies provide direct evidence that the loss of dACC is associated with decreased self‐awareness (Tow & Whitty, 1953). Therefore, increased GM structure in the identified dACC/mPFC, as the hub of DMN, might contribute to the processes such as negative bias in interpretation, generation and recall of information in people with high neuroticism, which are suggested to lead to common mental disorders (Ormel et al., 2013b).

This study has several limitations, some of which are inherent to the meta‐analytic approach. The inclusion of a relatively small number of studies on the GM correlates of neuroticism and peak coordinates‐based rather than raw statistical brain maps‐based meta‐analysis may result in a limited statistical power to detect associations between neuroticism and brain structure (Radua et al., 2014). Second, we focused solely on whole‐brain GM correlates of neuroticism as determined by VBM. Studies using other GM morphometric measurements (e.g., cortical thickness, surface area, and cortical folding) were not included due to the limited number of relevant studies and to reduce the methodological heterogeneity. With an increasing number of SBM studies investigating GM structure associated with neuroticism (Avinun et al., 2020; Hyatt et al., 2019; Owens et al., 2019; Privado et al., 2017; Riccelli et al., 2017), future meta‐analyses of corresponding studies are needed to reveal a more comprehensive neuroanatomical basis underlying neuroticism.

Given that previous studies found that significant associations of neuroticism with GM variations were modulated by gender (Blankstein et al., 2009; Nostro et al., 2017), and that the included studies covered subjects with a wide age range, subgroup meta‐analyses would be recommended. However, the small number of studies hampered us from conducting subgroup meta‐analyses. Moreover, since neuroanatomical variations of the dACC/mPFC have been associated with extraversion in a previous meta‐analysis (Lai et al., 2019), it is necessary to control for extraversion or to investigate interactive effects of extraversion and neuroticism on GM variations in this region. However, there is only one study in our meta‐analysis that treated the other four traits of FFM as nuisance covariates when identifying the GM correlates of neuroticism (Liu et al., 2013). Future studies are invited to examine other personality traits that may influence the structural neural basis of neuroticism.

More broadly, it has been argued that other personality models measure similar constructs to neuroticism, such as emotionality in HEXACO model (Lee & Ashton, 2004) and negative emotionality defined by Watson and Clark (1984) and Tellegen (1985). Notably, despite the relatively high correlation between neuroticism and emotionality (Gaughan, Miller, & Lynam, 2012) or negative emotionality (Church, 1994), these constructs reflect different conceptualizations especially when comparing respective assessment and facet scales (Widiger, 2009). In light of this, personality neuroscience approach could be used to better differentiate these personality traits in terms of the respective neural implementation. With emerging researches on this topic, a meta‐analytic comparison study of GMV is invited to reveal shared and distinct neuroanatomical substrates in these traits.

Last but not least, as neuropsychiatry studies have shown structural abnormalities in the dACC/mPFC in a range of mental disorders (Carlisi et al., 2017; Goodkind et al., 2015; Wise et al., 2017), and the prefrontal cortext and ACC has been acted as a target for the treatment of depressive disorders such as using transcranial magnetic stimulation (TMS; Hadas et al., 2019; Wu et al., 2020), a closer examination about the relationship between neuroticism, dACC/mPFC, and mental disorders will be needed in future studies.

## 5 CONCLUSIONS

In brief, our meta‐analysis identified for the first time robust neurostructural markers of neuroticism. Demographic factors including age and gender were not identified as potential confounders contributing to GM variations in the identified regions. Our findings may have clinical implications for the prevention and treatment of neuroticism. For instance, psychotherapy (e.g., mindfulness training; Stevens, Gauthier‐Braham, & Bush, 2018), and brain intervention programs (e.g., neurofeedback training, Sitaram et al., 2017; and TMS, Brunoni et al., 2017) targeting the dACC/mPFC might be useful for alleviating neuroticism, which may in turn reduce the risk of anxiety and mood disorders.

## CONFLICT OF INTEREST

The authors declare no competing interests.

## Supporting information


**Appendix** **S1:** Supporting information

## Data Availability

The data and code that support the findings of this study are available from the corresponding author upon reasonable request. The data and code sharing adopted by the authors comply with the requirements of the funding institute and the institutional ethics approval.
