# Supplementary material for: Gray matter structures associated with neuroticism: A meta‐analysis of whole‐brain voxel‐based morphometry studies
Source: Hum Brain Mapp. 2021 Mar 11;42(9):2706–21. doi: 10.1002/hbm.25395 (PMC8127153; doi:10.1002/hbm.25395)
Supplement: Supplementary file 1 — Appendix S1: Supporting information [file HBM-42-2706-s001.docx]

**Table S1. Results of Jackknife sensitivity analyses for areas showing neuroticism-GMV relations.**

| Discarded study | R/L dACC/mPFC |
| --- | --- |
| Cremers et al. (2011) | **Yes** |
| Du et al. (2015) | **Yes** |
| Hu et al. (2011) | **Yes** |
| Kapogiannis et al. (2013) | **Yes** |
| Koelsch et al. (2013) | **Yes** |
| Liu et al. (2013) | **Yes** |
| Lu et al. (2014) | **Yes** |
| Nostro et al. (2017) | **Yes** |
| Omura et al. (2005) | **Yes** |
| Taki et al. (2012) | **Yes** |
| Xu et al (2020) | **Yes** |
| Yang et al. (2016) | **Yes** |
| Zou et al. (2018) | **Yes** |

**Note: dACC, dorsal anterior cingulate cortex; mPFC, medial prefrontal cortex.**

**Table S2. Clusters showing significant between study heterogeneity**

| **R/L** | | **Clusters** | **Voxels** | **MNI coordinate** | **SDM-Z** | **P value** |
| --- | --- | --- | --- | --- | --- | --- |
| **R/L** | **median cingulate gyri/medial superior frontal gyrus** | | **407** | **4,28,38** | **1.532** | **0.000144482** |
| **R** | **lingual gyrus** | | **114** | **14,-66,2** | **1.371** | **0.000252903** |
| **R** | **Inferior frontal gyrus, orbital part** | | **33** | **40,38,-20** | **1.545** | **0.000103235** |
| **R** | **Rolandic operculum** | | **29** | **66,-8,12** | **1.·056** | **0.000686407** |
| **R** | **Middle frontal gyrus** | | **26** | **50,44,16** | **1.054** | **0.000691533** |

**Note: R, right; L, left; MNI, Montreal Neurological Institute.**

| **Table S3. Brain regions where gray matter structure is positively correlated with neuroticism in the meta-analysis with Kapogiannis et al.(2013) and Taki et al. (2012) excluded.** | | | | | | |
| --- | --- | --- | --- | --- | --- | --- |
| **Clusters** | **R/L** | **BA** | **Number of voxels** | **Peaks MNI coordinates (X, Y, Z)** | **SDM-Z** | **P value** |
| **dACC/mPFC** | **R/L** | **32** | **1467** | **8,48,22**  **4,40,26** | **1.865**  **1.831** | **~0**  **~0** |
| **Note: BA, Brodmann area; MNI, Montreal Neurological Institute; R, right; L, left;** **dACC, dorsal anterior cingulate cortex; mPFC, medial prefrontal cortex. Clusters were identified at voxelwise level of *p* < 0.005, z > 1 and cluster size > 10 voxels.** | | | | | | |
